# Genomics-based epidemiology of bovine *Mycoplasma bovis* strains in Israel

**DOI:** 10.1186/s12864-020-6460-0

**Published:** 2020-01-22

**Authors:** Yael Yair, Ilya Borovok, Inna Mikula, Rama Falk, Larry K. Fox, Uri Gophna, Inna Lysnyansky

**Affiliations:** 1 School of Molecular Cell Biology and Biotechnology, George S. Wise Faculty of Life Sciences, Tel Aviv University, Tel Aviv, Israel; 2 Mycoplasma Unit, Division of Avian Diseases, Kimron Veterinary Institute, POB 12, 50250 Beit Dagan, Israel; 3 Israel Dairy Board, Laboratory for Udder Health and Milk Quality, Caesarea, Israel; 4 Washington State University, Pullman, WA USA

**Keywords:** *Mycoplasma bovis*, Mastitis, Whole genome sequence, Single nucleotide polymorphism analysis

## Abstract

**Background:**

*Mycoplasma bovis* is an important etiologic agent of bovine mycoplasmosis affecting cattle production and animal welfare. In the past in Israel, *M. bovis* has been most frequently associated with bovine respiratory disease (BRD) and was rarely isolated from mastitis. This situation changed in 2008 when *M. bovis*-associated mastitis emerged in Israel. The aim of this study was to utilize whole genome sequencing to evaluate the molecular epidemiology and genomic diversity of *M. bovis* mastitis-associated strains and their genetic relatedness to *M. bovis* strains isolated from BRD in local feedlot calves and those imported to Israel from different European countries and Australia.

**Results:**

Phylogeny based on total single nucleotide polymorphism (SNP) analysis of 225 *M. bovis* genomes clearly showed clustering of isolates on the basis of geographical origin: strains isolated from European countries clustered together and separately from Australian and Chinese isolates, while Israeli isolates were found in the both groups. The dominant genotype was identified among local mastitis-associated *M. bovis* isolates. This genotype showed a close genomic relatedness to *M. bovis* strains isolated from calves imported to Israel from Australia, to original Australian *M. bovis* strains, as well as to strains isolated in China.

**Conclusions:**

This study represents the first comprehensive high-resolution genome-based epidemiological analysis of *M. bovis* in Israel and illustrates the possible dissemination of the pathogen across the globe by cattle trade.

## Background

**Fig. 1:**
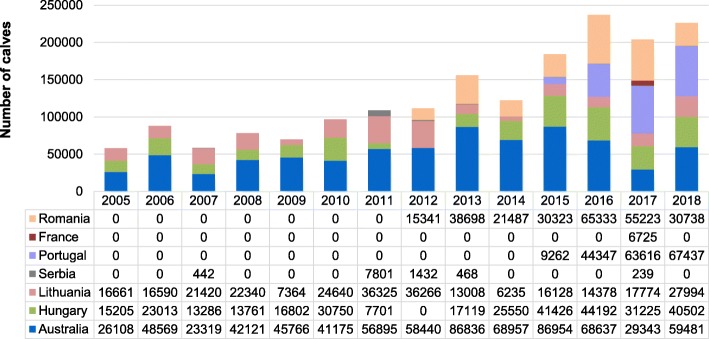
Number of calves imported to Israel from different European countries and Australia (2005–2018)

In Israel, dairy farming plays an important role within the agricultural sector with 20% of the output attributed to cattle and sheep (https://store.fil-idf.org/product/the-world-dairy-situation-2014-2/). The national cattle population is divided into 3 sectors: (a) pastured animals account for about 400 herds – approximately 60,000 heads; (b) feedlots, with about 500 herds – approximately 300,000 head, from which more than two thirds are calves imported from Australia and different European countries (Fig. 1); and (c) dairy farms with approximately 736 herds – around 250,000 head, 125,000 of which are milking cows.

The majority of Israeli dairy herds (about 76%) are located on small family-type farms with an average herd size of 116 cows, whereas 22% of the herds are located on large cooperative farms, with an average herd size of 457 cows; the rest of the farms (about 2%) belongs to the agricultural schools with an average herd size of 82 cows (https://www.halavi.org.il/). Dairy herds tend to be closed, with few to no animals comingling with cattle raised outside the herd. Unlike the dairy side of the cattle industry, feedlots have a high turnover of livestock. For example, according to the Israeli Veterinary Services (https://www.moag.gov.il/vet/Yechidot/inport%20export/knisa_yetsia_hayot_mahmad/yevu_mikne/Pages/default.aspx), the total number of imported animals, mainly calves, ranged from 57,974 in 2004 to 236,887 in 2016 with a total of 1,799,168 animals imported between 2005 and 2018. The countries from which Israel imported cattle include Australia (742,601 animals), Hungary (320,532), Romania (257,143), Lithuania (277,123), Portugal (184,662), Serbia (10,382) and France (6725) (Fig. 1).

On Israeli dairy farms, mastitis remains one of the most common and costly diseases where the large majority of clinical and subclinical bovine mastitis is caused by non-contagious pathogens (https://www.halavi.org.il/). Although there was a good success in control of the historically common contagious pathogens, *Staphylococcus aureus* and *Streptococcus agalactiae*, *Mycoplasma bovis* associated mastitis is now considered a significant contagious mastitis problem and accounts for approximately 3% of clinical milk submissions [1]. Since there are no efficacious antibiotics or vaccines for treatment or prevention of *M. bovis* mastitis, animal culling remains the recommended practice to control this disease, resulting in significant animal replacement costs to the producer [2]. According to the National Service for Udder Health and Milk Quality (NSUHMQ), over the last 15 years (2004–2018) a total of 95 dairy herds were positive for *M. bovis*: 18 of them were positive for *M. bovis* more than once ([1] and Lysnyansky et al., unpublished results). Until 2008, only sporadic cases of *M. bovis-*associated mastitis (0–3 positive herds per year; 2004–2007) were reported in Israel, usually originating from the same geographic region. In 2008, there was a widespread *M. bovis-*associated mastitis outbreak affecting 18 herds (total 61 cows). Since then, an average of 9 *M. bovis* positive herds are identified annually, more than 7 of which are newly infected ones (Lysnyansky et al., unpublished results). Mastitis due to *M. bovis* has also increased over the last decade in many European countries and outbreaks have been reported in Austria [3, 4], Denmark [5], the Netherlands [6] and Switzerland [7]. Moreover, countries such as Norway and New Zealand, which were previously free of *M. bovis*, became positive ([2]; https://www.mpi.govt.nz/dmsdocument/29015-m-bovis-timeline-fact-sheet). Clearly *M. bovis*-associated mastitis appears to be an emerging global problem [8]. Additionally, *M. bovis* is a component of the bovine respiratory disease complex (BRD) and a significant concern to livestock producers around the world [9, 10].

In the past, several techniques allowing genetic differentiation of *M. bovis* have been applied in order to decipher the molecular epidemiology of this pathogen. For example, multiple locus variable number tandem repeat (VNTR) analysis demonstrated identity between *M. bovis* isolates from Israeli dairy cows and calves imported from Australia and suggested possible introduction of strains from imported animals into local dairy herds [11]. In addition, multi-locus sequence typing (MLST) [12] of 57 Israeli mastitis associated *M. bovis* isolates (2004–2014) revealed the presence of a dominant genotype (ST10), present in 60% of the tested strains [1]. Interestingly, ST10 was also identified as the dominant genotype in a cohort of Chinese strains isolated from BRD and pneumonia [13]. China, like Israel, imports cattle from Australia which raises a question of possible spread of *M. bovis* strains across international boundaries by animal movement. The circulation of dominant *M. bovis* clones or lineages (other than ST10) has also been observed in several European countries [14–16] and a possible link between the appearance of new dominant genotypes of *M. bovis* and the emergence of severe clinical mastitis cases has been suggested [14].

Single nucleotide polymorphism (SNPs) analysis based on whole genome sequencing (WGS) (referred to here as SNP-WGS) has a higher level of discriminatory power than the conventional molecular typing methods mentioned above, facilitating its implementation for diagnosis, epidemiological investigations, comparative and evolutionary genetic studies as well as for routine surveillance [17]. It is a robust tool for studying closely related strains of pathogenic bacteria such as mycobacteria and drug-resistant *Escherichia coli*, *Salmonella enterica* serotype Typhimurium, *Staphylococcus aureus*, *Clostridioides difficile*, *Clostridium perfringens* and many other species [18–23]. Notably, the WGS-SNP analysis was applied for genetic characterization of Australian *M. bovis* isolates and showed the circulation of a single strain throughout the country [24]. The aim of this study was to utilize WGS-SNP to evaluate the molecular epidemiology of Israeli *M. bovis-*mastitis strains and their genetic relatedness to *M. bovis* strains isolated from BRD of local feedlot calves and those imported to Israel from Australia and various European countries.

## Results

### Phylogenetic relationships of local *M. bovis* isolates isolated from mastitis

**Fig. 2:**
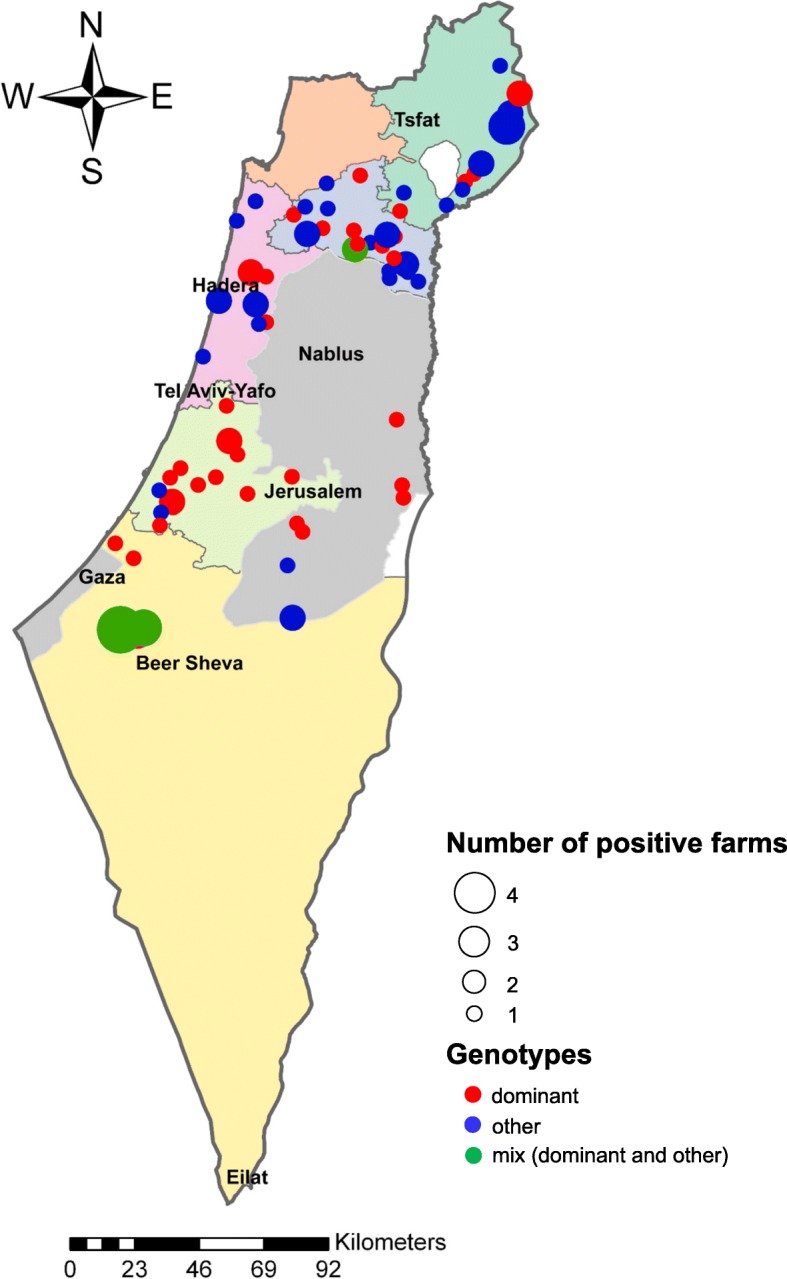
Geographical distribution of dairy farms from which *M. bovis* mastitis-associated isolates (1994–2017) that were included in this study were collected. The map was prepared by using ArcGIS Pro 2.2.4 software (https://support.esri.com/en/products/desktop/arcgis-desktop/arcgis-pro/2-2-4). The radius of each circle represents the number of *M. bovis* mastitis episodes in that farm and/or the number of *M. bovis*-positive farms within the same settlement. Dominant mastitis – associated and other genotypes were colored by red and blue, respectively, while mixed genotype (dominant and other) was colored by green. The regional veterinary districts and Palestinian authority are showed by different colors

**Fig. 3:**
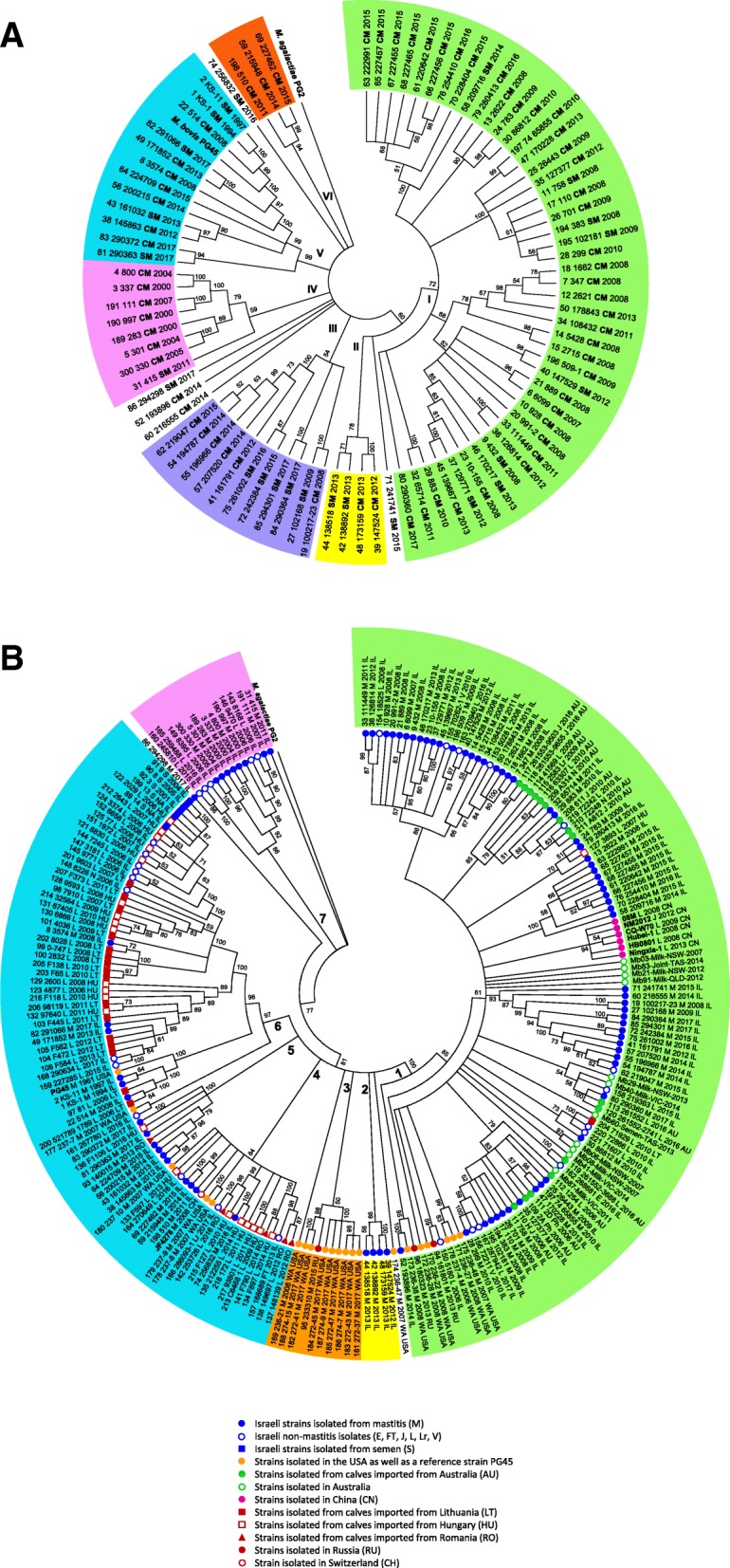
Total-genome SNP-based phylogenetic trees of *M. bovis*. The phylogenetic trees were constructed using MEGAX [25] with Maximum Likelihood phylogeny method. Values on branches display support values (500 bootstraps). Branches corresponding to partitions that were reproduced in less than 50% of bootstrap replicates were collapsed. **a** Phylogeny of 89 *M. bovis* isolates isolated from mastitis on local dairy farms (1994–2017). *M. bovis* PG45 and *M. agalactiae* PG2 type strains were included into comparison and indicated by bold. The clades (I-VI) are marked and represented by different color. The designation of the isolates includes serial number or sequencing identifier (for isolates sequenced in this study), name of the isolate, type of mastitis (clinical (CM) or subclinical (SM)) and year isolation. **b** Phylogeny of 225 *M. bovis* isolates. Strains for which the complete genomes were used are shown by bold. Country’s origin of the strains is indicated by colored symbols. The different clades (1–7) are marked and represented by different color. The clades mainly contained “AU” and “EU”-related isolates are marked by green and blue colors, respectively. The designation of the isolates includes serial number or sequencing identifier (for isolates sequenced in this study), name of the isolate, anatomical site of isolation, year and country of isolation. For additional information about particular strain, see Additional file 1: Table S1. E – eye; FT – fetal tissue; J – joint; L – lung; Lr – larynx; M – milk; N – nasal cavity; P – pharynx; S – semen; V – vulva

To infer the relationships among *M. bovis* isolates, isolated from mastitis between 1994 and 2017 in Israel (Fig. 2), WGS-SNP phylogenetic analysis was performed. The phylogenetic tree generated based on the total-genome SNP matrix (total 28,912 SNPs) revealed 6 main clades (Fig. 3a; the same inferred tree with scale bar and bootstrap values can be found in Additional file 4). Clade I is the largest and dominant clade which contains 46 *M. bovis* isolates, most of which (*n* = 43) have been previously typed by MLST as ST10 (Lysnyansky et al., unpublished results, [1, 12]). Clade II and III include 4 and 11 isolates, respectively; some of the isolates related to these clades are likely to be epidemiologically linked (Additional file 1: Table S1). Clade IV consists of 8 isolates, with STs 23–25 and 35–37 [1, 12]. Most of the isolates belonging to this clade were isolated from sporadic cases of mastitis prior to the 2008 mastitis outbreak. Clade V contains 13 isolates, 6 of which belong to ST39 (Lysnyansky et al., unpublished results, [1]). This group includes the reference type strain *M. bovis* PG45 which clusters with three local isolates, two of which (KS-1 and KS-11) were isolated from sporadic cases of mastitis in 1994 and 1997, respectively and one (514) was isolated in 2008 during a mastitis outbreak (Fig. 3a). Clade VI consists of 3 isolates and all of them were typed previously as ST8 (Lysnyansky et al., unpublished results, [1]) (Fig. 3a).

### Population structure of *M. bovis* strains isolated from local and imported cattle

In order to decipher the complexity of *M. bovis* population structure in Israel, a total of 225 isolates and *M. agalactiae* PG2, used as outgroup, were subjected to WGS-SNP phylogenetic analysis (Additional file 1: Table S1 and Fig. 3b; the same inferred tree with scale bar and bootstrap values can be found in Additional file 5). Total SNP count for 226 isolates resulted in 37,828 SNPs. In this comparison a clear separation between cohorts of isolates isolated from calves imported from European countries and isolates isolated from China and Australia (original as well as “imported” isolates) was evident, while Israeli isolates were identified in both groups. Based on the total SNP matrix, 7 main clades (1–7) were obtained (Fig. 3b). Clade 1 contains 115 *M. bovis* isolates separated into several lineages with a number of isolates ranged from 2 to 24 (Fig. 3b). Isolates in this clade mainly originated from Australia (original Australian isolates (*n* = 11/11) and isolates isolated from calves that were imported from Australia to Israel (*n* = 16/16)), China (*n* = 6/6), the USA (*n* = 6/20) and other countries (*n* = 4) (Fig. 3b). In addition, clade 1 includes 72 Israeli isolates from which 60 and 12 were isolated from mastitis and other clinical conditions, respectively. Notably, all local mastitis-associated isolates branched previously in clades I and III (Fig. 3a) were identified in different lineages of clade 1 (Fig. 3b). Out of all local isolates found in clade 1, 73.6% typed as ST10 by MLST developed recently by Rosales et al., [12]. Moreover, 87.5% of isolates imported from Australia to Israel and 66.6% of mastitis isolates identified in the USA and are related to clade 1 also belong to ST10 (data not shown). While all Chinese isolates clustered together in separate lineage, the original Australian *M. bovis* isolates were found either as singletons (Mb03, 83, 21, 91, 60 and 41), or clustered in a separate lineage (Mb06 and Mb08) or clustered together with local and “AU-imported” isolates (Mb29, Mb40 and Mb87) (Fig. 3b).

In contrast to the origin of the isolates identified in clade 1, isolates that originated from European countries were mainly distributed across two clades 4 and 6 (Fig. 3b). For example, clade 4 includes 22 isolates that originated from Hungary (*n* = 7/22), Romania (*n* = 4/5), the USA (*n* = 3/20) and Switzerland (*n* = 1/1) as well as 7 local isolates, while clade 6 contains 51 *M. bovis* isolates that originated from Hungary (*n* = 13/22), Lithuania (*n* = 16/17), and the USA (*n* = 2/20 and *M. bovis* PG45 type strain) as well as 20 local isolates (Fig. 3b). Notably, clade 6 contains 5 local isolates identified from semen (14, 13, 3, 8 and 9), which clustered together with some local and “HU-imported” isolates associated with pneumonia. No 100% identity was identified between semen and mastitis-related isolates used in this study. The mastitis related isolates found in clades 4 and 6 have been previously clustered in clades VI and V, respectively (Fig. 3a). Clade 5 (*n* = 8) also includes 2 isolates originated from the EU countries such as Romania and Hungary (Fig. 3b). These isolates are clustered together with 6 local mastitis-related isolates found previously in clade V (Fig. 3a). The rest of total SNP matrix-related clades (2, 3 and 7) are relatively small and encompasses 4, 9 and 12 isolates, respectively. While most of the isolates found in clades 2 and 7 are local ones, most of the isolates related to clade 3 were isolated in the USA (Fig. 3b).

In this study, no statistically significant association was observed between the clinical manifestation of disease and a particular SNP. Although we identified 930 SNPs as being significantly enriched in mastitis isolates, yet when controlling for the phylogenetic relatedness of the strains, none of them reached statistical significance (data not shown).

## Discussion

In this study, we applied whole genome sequencing and subsequent WGS-SNP analysis to resolve *M. bovis* intraspecies relationships in Israel and to derive epidemiological conclusions from the population structure of this bovine pathogen. First, the data obtained by total SNP analysis clearly show clustering of isolates on the basis of geographical origin. Indeed, strains isolated from European countries clustered together and separately from Australian and Chinese isolates, while Israeli isolates were found in the both groups. A similar distribution of geographically distant isolates was previously observed by VNTR [11] and MLST [12]. Notably, *M. bovis* type strain PG45, isolated in 1961 in the USA [26], grouped with Israeli and European rather than with American *M. bovis* strains recently isolated from mastitis in Washington state (Additional file 1: Table S1 and Fig. 3b). This finding underlines the hypothesis of common ancestors between *M. bovis* PG45 and European isolates, probably as results of cattle trade between two continents in the past and confirms previous reports demonstrating close linkage between European *M. bovis* isolates and PG45 that had been obtained by AFLP [27] and MLST [12].

Second, total SNP analysis demonstrated the presence of a dominant genotype among Israeli *M. bovis* strains associated with mastitis with 51.6% (46/89) of the strains related to clade I (Fig. 3a). The first mastitis isolate (6099) with this genotype was identified on a dairy farm in 2007 prior to the mastitis outbreak of 2008 (Additional file 1: Table S1). It differed markedly from *M. bovis* strains isolated from sporadic cases of mastitis before 2008 (Fig. 3). The circulation of a dominant *M. bovis* clone or lineage has also been observed in several European countries. For example, Bürki et al., [14] demonstrated that a shift in circulating Swiss and Austrian *M. bovis* isolates occurred in 2007: isolates collected since 2007 in both countries belonged to lineage I, while all Swiss isolates recovered before 2007 clustered in lineage II. The authors suggested a link between appearance of new genotypes of *M. bovis* and the emergence of severe clinical mastitis cases. The presence of a dominant lineage of *M. bovis* was also observed in Denmark where *M. bovis* strains identified from recent outbreaks (2011–2014) clustered together and differed from strains isolated in the outbreaks of 1984 or 1987 and from later sporadic isolates [15]. The emergence of a new dominant *M. bovis* subtype was also shown in France [16]. However, in France, the emerging ST was mostly associated with pneumonia and no increase in cases of mastitis has been observed. Interestingly, *M. bovis* clonal homogeneity was also identified in the cohort of Chinese isolates (*n* = 44), 97.7 and 95.5% of which were typed as MLST-ST10 by two different MLST schemes [13]. In Australia, circulation of a single *M. bovis* strain was detected by WGS-SNP analysis of 75 isolates (2006–2015) isolated from various clinical presentations with a maximum of 50 SNPs observed between any two isolates [24].

Third, the results obtained in our study also confirmed the genetic similarity among local *M. bovis* mastitis isolates related to the dominant clade I as well as to clade III, strains isolated from calves imported to Israel from Australia and the original Australian and Chinese strains (Fig. 3). The possible explanation of the widespread distribution of this clone is global livestock movement since both Israel and China import cattle from Australia (https://www.beefcentral.com/live-export/australias-10-largest-cattle-exports-markets-in-2018/).

Recently MLST analysis [28] of *M. bovis* isolates isolated in Japan revealed that local isolates belonging to the ST10 subgroup first emerged in 2014, and their abundance has been increasing in recent years [29]. Notably, the import of breeding stock for Japanese dairy cattle from Australia became an almost exclusive source in the last 14 years [29]. Transmission and spread of *M. bovis* via cattle trade is not a new phenomenon and can be exemplified by the situation that occurred in Northern Ireland (NI). Reportedly, *M. bovis* was not present in NI prior to the relaxation of trade regulations more than 25 years ago. Yet after joining the European Union, imported cattle with *M. bovis* entered NI resulting in cases of *M. bovis*-associated pneumonia in calves [30]. Since that time *M. bovis* has become a significant contributor to calf pneumonia in NI [31]. Although the situation in NI is an example of the introduction of *M. bovis* into what appears to have been a completely naïve population, the introduction of new *M. bovis* strains into endemic area may also result into *M. bovis-*associated outbreaks.

While introduction of the “Australian” clone to Israel is easy to explain, it is harder to understand how such clone has disseminated among dairy farms. In general, most of the Israeli dairy farms maintain a ‘closed herd’ policy and rarely introduce new cows from other farms or import calves for feeding. With several exceptions (see Additional file 1: Table S1), the source, risk factors as well as an epidemiological link among the mastitis events on Israeli dairy farms are largely unknown. However, the fact that about 82% of *M. bovis* positive herds identified during the 2008 mastitis outbreak and tested in this study possessed the dominant clade I related genotype (Fig. 3a) may point to a common source of infection or/and epidemiological link. One possible explanation of such a situation may be transmission via artificial insemination (AI) as has been previously described in Finland [32]. In Israel, *M. bovis* was cultured from several semen lots collected before 2008, but neither VNTR [11], MLST [1, 12] nor WGS-SNP analysis performed in this study revealed identity between semen strains and the dominant genotype (Fig. 3b). However, some semen isolates showed SNP similarity to several local as well as HU-"imported” pneumonia-associated isolates (Fig. 3b). Some of the calves, raised for AI are purchased from local farms. These calves could have been the reservoir for the disease as clinically healthy asymptomatic carriers that intermittently shed *M. bovis*. Moreover, many feedlots import calves for feeding and this fact can explain the similarity between cohorts of semen-related isolates and “HU-imported” isolates (Fig. 3b).

It is probable that the *M. bovis* mastitis-dominant lineage, identified in this study, harbors some specific virulence characteristics, which contribute to its predilection to mammary gland results in domination of this clone on local dairy farms. However, until now, no data demonstrating a clear difference in tissue or organ specificity have been observed among bovine *M. bovis* strains and we also did not find statistically significant association between clinical manifestation of disease and a particular SNP (data not shown). In addition, genome-based characterization of *M. bovis* virulence factors performed on genetically-similar Australian *M. bovis* strains failed to identify genes which are specific to different geographical location or anatomical site [24]. In another study, several virulence-related genes were deleted or have accumulated mutations and indels in three *M. bovis* attenuated clones when comped to the parental *M. bovis* HB0801 strain [33]. The impact of such changes on *M. bovis* virulence remains to be investigated. In France, the selection and dissemination of the dominant *M. bovis* clone was linked to increased antibiotic resistance [34], which is often associated with decreased virulence and fitness [35]. Interestingly, despite the highly contagious nature of *M. bovis*, mastitis associated with this species in Israel does not tend to persist upon emergence in dairy herds. Indeed, on the average 82% of the positive herds identified annually are newly infected (Lysnyansky et al., unpublished results). Rapid clearance of *M. bovis* mastitis in dairy herds has been reported previously [36]. The possible explanation of this situation may be effective application of the prevention strategies on the farms, the circulation of a putative low-virulence *M. bovis* strains and/or spontaneous recovery by infected cattle. Further studies should evaluate the pathogenicity and infectivity of the dominant clone compared to previously studied lineages. Moreover, the fact that phylogenetically closely-related Chinese *M. bovis* isolates cause mostly respiratory disease and pneumonia highlights the possibility that other factors (host, environment or husbandry practices) may play a role in disease development.

In summary, *M. bovis* mastitis appears to be an emerging worldwide problem. Therefore, the data obtained in this study may contribute to further understanding of the global epidemiology and surveillance of this pathogen and may be of crucial importance in development of *M. bovis* control strategies.

## Conclusions

*M. bovis* mastitis appears to be an emerging worldwide problem, therefore the data obtained in this study may contribute to further understanding of the global epidemiology and surveillance of this pathogen and may be of crucial importance in development of *M. bovis* control strategies. In addition, the availability of multiple genome sequences may provide the basis for further studies on evolution, population and structure-function pathobiology analyses of this pathogen.

## Methods

### *Mycoplasma bovis* strains used for WGS in this study

A total of 221 *M. bovis* isolates were selected in order to achieve two main populations: mastitis-related isolates isolated on local dairy farms and respiratory related isolates isolated from local feedlots and calves imported to Israel from different European countries and from Australia (Additional file 1: Table S1). Most of *M. bovis* isolates (*n* = 201) were isolated in the Mycoplasma unit, Kimron Veterinary Institute, Israel, while 20 mastitis-related isolates were isolated in Washington State University, WA, USA. Imported animal of origin was sourced from the country indicated (Australia, Lithuania, Hungary and Romania; Additional file 1: Table S1). The sampling of these animals were performed either in quarantine stations in Israel or in the pathology department of KVI, in case they died during transportation to Israel. All *M. bovis* isolates were collected, cultured and preserved as part of standard diagnostics. Consequently, no permission or ethical approval for isolate collection was needed.

Isolates from the nasal cavity or semen were isolated from healthy animals, while isolates from the joint, lung, pharynx, larynx and vagina were isolated from clinical cases (Additional file 1: Table S1). Milk samples were obtained either from clinical or subclinical mastitis. Clinical mastitis was defined when any visual changes in the milk (color, fibrin clots) or in the udder (swelling, heat, pain, redness) were observed while subclinical mastitis was defined as a rise in the somatic cell count (≥200,000 SCC) without any visual changes in the milk and udder.

### Growth conditions, DNA extraction and PCR analysis for species verification

Isolates were propagated at 37 °C in standard *M. bovis* broth medium [37] supplemented with 0.5% (wt/vol) sodium pyruvate and 0.005% (wt/vol) phenol red [38], at pH 7.8. *M. bovis* colonies were initially identified by direct immunofluorescence (IMF) with species-specific conjugated antiserum [39]. Mixed cultures were diluted to IMF homogeneity by microscopic selection of target colonies. All isolates were filter-cloned at least once. DNA was extracted from 10 ml logarithmic-phase cultures using the DNeasy blood and tissue kit (Qiagen, GmbH, Hilden, Germany) following the manufacturer’s instructions. DNA concentration and purity of the DNA samples were assessed by NanoDrop ND-1000 spectrophotometer (Thermo Scientific). DNA samples were analyzed using *M. bovis* specific PCR [40] as well as universal *Mycoplasma* spp. PCR [41, 42]. The amplicons obtained by the universal PCR was then confirmed as *M. bovis* via Sanger Sequencing (Hylab, Rehovot, Israel).

### Whole genome next generation sequencing and assembly

Of these *M. bovis* 221 isolates, 188 were sequenced in this study (see below), while 33 that were isolated in Israel between 2000 and 2011, have been previously sequenced by Wellcome Trust Sanger Institute (UK) and deposited in NCBI (Bio project PRJEB3408). DNA samples from 188 isolates were sequenced using an Illumina NextSeq500 platform at the Chicago Sequencing Center of the University of Illinois, generating 2 × 150 paired-end reads. Sequencing was performed in 4 separate runs. Reads from all runs were concatenated to a single file. The sequencing depth ranged from ~ 1,800,000 reads to ~ 8,200,000 per sample, with average coverage of around 300x. Two isolates presented low coverage and were excluded from further analysis. Adaptors and low-quality sequences were trimmed using Trimmomatic-0.36 [43].

Assembly of draft genomes was performed using the SPAdes 3.9.1 assembler [44] in –careful mode, with k-mer length of 127. Assembly metrics (average coverage, N50, number of contigs and assembly size) are presented in Additional file 2: Table S2. The assembly quality and completeness were assessed using QUAST [45] and the CheckM pipeline [46]. Eleven draft genomes were later removed from further analysis due to low assembling quality as well as sequence contamination, possibly as a result of mixed infection.

Finally, a total of 217 genomes (175/188 sequenced in this study, 31/33 sequenced by Wellcome Trust Sanger Institute (UK) and 11 genomes from Parker et al., [24]) were successfully assembled. Contigs composed of less than 1000 nucleotides were excluded from the final assemblies. Final draft genomes contain an average of 90 contigs per genome. The genomes described in this manuscript have been deposited in the National Center for Biotechnology Information (NCBI)‘s under the project accession number PRJNA564939 and their accession numbers have been provided in Additional file 1: Table S1.

### SNP calling

Single Nucleotide Polymorphism (SNP) detection was performed using kSNP3.0, a tool for SNP detection and phylogenetic analysis of genomes without the need for genome alignment or reference genome, as previously described [47]. Briefly, kSNP3.0 allows the detection of pan-genome SNPs in a set of genome sequences for further phylogenetic analysis and investigation. Using a small set of annotated genomes from NCBI database, SNP calling with annotations was performed on all genomes with different genome combinations (see Result section). The complete genomes of *M. agalactiae* PG2 type strain (NC_009497 [48];), *M. bovis* PG45 type strain (NC_014760 [49];), Chinese strains CQ-W70 (NZ_CP005933), HB0801 (NC_018077 [50];), Hubei-1 (NC_015725 [51];), 08 M (NZ_CP019639), Ningxia-1 (NZ_CP023663), NM2012 (NZ_CP011348) and *M. bovis* strain JF4278 (NZ_LT578453) were retrieved from GeneBank and included in the analyses. The kSNP run was performed with – core flag, and m-mer size of 31 (selected after optimization with Kchooser, one of Ksnp3.0 utilities). Total SNPs were all SNPs detected in the analysis.

The SNP matrices generated were used to create phylogenetic trees. The appropriate substitution model was selected using MEGA-X, with General Time Reversible model (GTR) being the best substitution model with BIC score of 459,247.6 ([25] (see Additional file 3: Table S3) for local mastitis isolates analysis, and with GTR with Gamma distribution of 4 for the analysis of all strains (BIC score of 871,253.1). A parallel analysis for SNPs detection with reference strain *M. bovis* PG45 (Additional files 6 and 7) was done using CSI phylogeny web server [52], with the following parameters: minimum depth at SNP position set at 10×, relative depth at SNP position set at 10×, minimum distance between SNPs set at 10, minimum SNP quality set at 30, minimum mapping quality set at 25 and a minimum Z-score of 1.96 corresponding to a *P* value of 0.05. The phylogenetic tree was constructed by using the Maximum-Likelihood method, and bootstrapped 500 times to assess support for the different branches using MEGA-X [25].

### *M. bovis* populations used for the comparisons

In the first comparison (Fig. 3a and Additional files 4 and 6), the phylogenetic relationships of local *M. bovis* mastitis-associated isolates (*n* = 89) was investigated (Additional file 1: Table S1; Numbers 1–80, 175–183). The samples were isolated from clinical (*n* = 65) and subclinical mastitis cases (*n* = 24) between the years 1994–2017 (Fig. 2). In addition, genomes of *M. bovis* PG45 and *M. agalactiae* PG2 type strains were included. For total-genome-based SNP analysis in comparison to a reference genome *M. agalactiae* was excluded (Additional file 6).

The second comparison examined the phylogenetic relationships among *M. bovis* isolates isolated from local and imported cattle as well as isolates isolated in Australia and China (Fig. 3b, Additional files 5 and 7). A total 226 isolates were included in this comparison from which 8 genomes are the complete genomes of *M. bovis* strains (see above), 11 genomes (Mb03, Mb06, Mb08, Mb21, Mb29, Mb40, Mb41, Mb60, Mb83, Mb87, Mb91) are original Australian *M. bovis* strains [24] and one genome is *M. agalactiae* type strain PG2 [48] used as outgroup (Additional file 1: Table S1, N207–226). For total-genome-based SNP analysis in comparison to a reference genome *M. agalactiae* was excluded (Additional file 7). The remaining 206 isolates (Additional file 1: Table S1, N1-206) sequenced in this study (*n* = 175) or by Sanger (*n* = 31) can be divided into the following categories: (i) mastitis associated samples (*n* = 89) isolated from local cows between the years 1994–2017 (Fig. 2); (ii) samples (*n* = 59) isolated in quarantine stations from calves originated from Lithuania (*n* = 17, 2006–2013), Australia (*n* = 16, 2006–2016), Hungary (*n* = 22, 2006–2016) and Romania (*n* = 4, 2012–2016); (iii) samples collected from local feedlots (*n* = 28, 2006–2017) and one cow (2010); (iv) samples isolated from frozen semen samples of healthy bulls (*n* = 5, collected between 2001 and 2008, but isolated between 2008 and 2009) and (v) samples isolated from milk of cows (*n* = 24) originating from various geographic locations including Romania (*n* = 1, 2013), Russia (*n* = 3, 2013–2015) and the USA (*n* = 20, 2006–2017). The group of mastitis-related isolates (*n* = 113) included *M. bovis* isolated from clinical (*n* = 82) and subclinical mastitis cases (*n* = 31). The group of non-mastitis isolates (*n* = 93) included those isolated from cases of pneumonia/BRD (*n* = 76), arthritis (*n* = 5), other clinical conditions (*n* = 4) as well as from healthy animals (*n* = 8) (Additional file 1: Table S1).

## Supplementary information

**Additional file 1:** **Table S1.** List of *M. bovis* strains used in this study. ^#^ Alternative IDs for isolates sequenced by Wellcome Trust Sanger Institute (UK) and used in our study. ^1–14^ The same superscript numbers indicate that *M. bovis* isolates were isolated on the different farms, located in the same settlement. ^A-I^ The same superscript characters indicate that *M. bovis* isolates were isolated on the same farm or from the same shipment. * There is an epidemiological link among the isolates. NR – not relevant; NA – not available.

**Additional file 2: Table S2.** Assembly results. Assembly results of 175 *M. bovis* isolates sequenced in this study (N1–175) and 31 Israeli isolates (N176–206) previously sequenced by Wellcome Trust Sanger Institute (UK) and deposited in NCBI (Bio project PRJEB3408).

**Additional file 3: Table S3.** Nucleotide substitution model selection.

**Additional file 4.** WGS-SNP-based phylogenetic tree presented in Fig. 3a allows to see the scale bar and branches of support. This file can be opened using MEGA software [25] or via public available servers, e.g. the NCBI Tree Viewer (https://www.ncbi.nlm.nih.gov/tools/treeviewer/), ETE Toolkit (http://etetoolkit.org/treeview/) etc.

**Additional file 5.** WGS-SNP-based phylogenetic tree presented in Fig. 3b allows to see the scale bar and branches of support. This file can be opened using MEGA software [25] or via public available servers, e.g. the NCBI Tree Viewer (https://www.ncbi.nlm.nih.gov/tools/treeviewer/), ETE Toolkit (http://etetoolkit.org/treeview/) etc.

**Additional file 6. **WGS-SNP-based phylogenetic analysis of local *M. bovis* mastitis-associated isolates (*n* = 89) performed in comparison to a reference genome *M. bovis* PG45 as described in Materials and Methods. The phylogenetic tree was constructed by using the Maximum-Likelihood method, and bootstrapped 500 times to assess support for the different branches using MEGA-X [25]. The designation of the isolates includes serial number or sequencing identifier (for isolates sequenced in this study), name of the isolate, type of mastitis (clinical (CM) or subclinical (SM)) and year isolation. For additional information about particular strain, see Additional file 1: Table S1. Total SNP count resulted in 16,910 SNPs. Comparison between phylogenetic trees obtained by total-genome-based SNP analysis with (Additional file 6) and without (Fig. 3a) comparison to the reference genome revealed almost the same intergroup clustering of the isolates. However, the clade distribution of isolates was slightly different between these two comparisons. This file can be opened using MEGA software [25] or via public available servers, e.g. the NCBI Tree Viewer (https://www.ncbi.nlm.nih.gov/tools/treeviewer/), ETE Toolkit (http://etetoolkit.org/treeview/) etc.

**Additional file 7. **WGS-SNP-based phylogenetic analysis of all *M. bovis* isolates performed in comparison to a reference genome *M. bovis* PG45 as described in Materials and Methods. The phylogenetic tree was constructed by using the Maximum-Likelihood method, and bootstrapped 500 times to assess support for the different branches using MEGA-X [25]. The designation of the isolates includes serial number or sequencing identifier (for isolates sequenced in this study), name of the isolate, anatomical site of isolation, year and country of isolation. For additional information about particular strain, see Additional file 1: Table S1. E – eye; FT – fetal tissue; J – joint; L – lung; Lr – larynx; M – milk; N – nasal cavity; P – pharynx; S – semen; V – vulva. Total SNP count resulted in 7203 SNPs. Phylogenetic tree received by comparison of 224 *M. bovis* isolates to the reference genome *M. bovis* PG45 supported a separation on the geographical origin. This file can be opened using MEGA software [25] or via public available servers, e.g. the NCBI Tree Viewer (https://www.ncbi.nlm.nih.gov/tools/treeviewer/), ETE Toolkit (http://etetoolkit.org/treeview/) etc.

## Data Availability

This Whole Genome Shotgun project has been deposited at DDBJ/ENA/GenBank under the accession XXXX00000000. Genome assembling accession numbers are written in Additional file 1: Table S1 (project no. PRJNA564939). The biomaterial will be available under request.

## Abbreviations

BRD: Bovine respiratory disease
*M. bovis*: *Mycoplasma bovis*
MLST: Multi-locus sequence typing
NSUHMQ: National Service for Udder Health and Milk Quality
SCC: Somatic cell count
SNP: Single nucleotide polymorphism
ST: Sequence type
VNTR: Variable number tandem repeat
WGS: Whole genome sequencing
